# Impact of Chronic Kidney Disease on the Presence and Severity of Aortic Stenosis in Patients at High Risk for Coronary Artery Disease

**DOI:** 10.1186/1476-7120-9-31

**Published:** 2011-11-16

**Authors:** Chiaki Masuda, Kaoru Dohi, Yuko Sakurai, Yuri Bessho, Harumi Fukuda, Shinobu Fujii, Tadafumi Sugimoto, Masaki Tanabe, Katsuya Onishi, Katsuya Shiraki, Masaaki Ito, Tsutomu Nobori

**Affiliations:** 1 Central Laboratory, Mie University Graduate School of Medicine; 2 Department of Molecular and Laboratory Medicine, Mie University Graduate School of Medicine; 3 Department of Cardiology and Nephrology, Mie University Graduate School of Medicine; 4 Department of Gastroenterology, Mie University Graduate School of Medicine

**Keywords:** Echocardiography, Aortic valve area, Coronary artery disease, Chronic kidney disease

## Abstract

**Objective:**

We evaluated the impact of chronic kidney disease (CKD) on the presence and severity of aortic stenosis (AS) in patients at high risk for coronary artery disease (CAD).

**Methods:**

One hundred and twenty consecutive patients who underwent invasive coronary angiography were enrolled. Aortic valve area (AVA) was calculated by the continuity equation using transthoracic echocardiography, and was normalized by body surface area (AVA index).

**Results:**

Among all 120 patients, 78% had CAD, 55% had CKD (stage 3: 81%; stage 4: 19%), and 34% had AS (AVA < 2.0cm^2^). Patients with AS were older, more often female, and had a higher frequency of CKD than those without AS, but the prevalence of CAD and most other coexisting conventional risk factors was similar between patients with and without AS. Multivariate linear regression analysis indicated that only CKD and CAD were independent determinants of AVA index with standardized coefficients of -0.37 and -0.28, respectively. When patients were divided into 3 groups (group 1: absence of CKD and CAD, n = 16; group 2: presence of either CKD or CAD, n = 51; and group 3: presence of both CKD and CAD, n = 53), group 3 had the smallest AVA index (1.19 ± 0.30*# cm^2^/m^2^, *p < 0.05 vs. group 1: 1.65 ± 0.32 cm^2^/m^2^, and #p < 0.05 vs. group 2: 1.43 ± 0.29* cm^2^/m^2^) and the highest peak velocity across the aortic valve (1.53 ± 0.41*# m/sec; *p < 0.05 vs. group 1: 1.28 ± 0.29 m/sec, and #p < 0.05 vs. group 2: 1.35 ± 0.27 m/sec).

**Conclusion:**

CKD, even pre-stage 5 CKD, has a more powerful impact on the presence and severity of AS than other conventional risk factors for atherosclerosis in patients at high risk for CAD.

## Introduction

Calcific aortic valve disease is associated with increased cardiovascular risk [1,2]. There are significant similarities in clinical risk factors and histopathological alterations between calcific aortic valve disease and coronary atherosclerosis [3]. It is also widely recognized that both cardiac and renal diseases are commonly present in the same patient, and the presence of chronic kidney disease (CKD) further accelerates and amplifies the process of aortic valve calcification via multiple pathways, such as altered mineral metabolism, inflammation, oxidative stress, and pressure and volume overload [4,5]. Previous studies have demonstrated an association between end-stage renal disease and calcific cardiovascular disease, especially in patients on dialysis [6-9]; however, less is known about the impact of early stage CKD on the prevalence and severity of calcific aortic valve disease. Accordingly, we tested the hypothesis that patients with early stage CKD not yet on dialysis who are at high risk for coronary atherosclerosis have an increased risk for developing calcific aortic valve disease.

## Patients and Methods

### Study Population

We prospectively evaluated the presence and severity of calcific aortic valve disease in a consecutive series of 146 patients who underwent coronary angiography between September 2009 and March 2010 because of suspected coronary artery disease or for follow up angiography after coronary angioplasty or coronary artery bypass graft surgery. We excluded 1 patient with rheumatic heart disease, 1 patient with severe aortic regurgitation due to infective endocarditis, 1 patient with a bicuspid aortic valve, and 8 patients with prosthetic valves due to specific pathophysiologic conditions that affect the aortic valve area other than "calcific" aortic stenosis. One patient with known severe aortic stenosis who was referred to our hospital for preoperative evaluation, not for the suspicion of coronary artery disease, was also excluded. Nine patients with end-stage renal disease treated with hemodialysis were not included in the present study to exclude the confounding effect of hemodialysis on the progression of aortic valve sclerosis. Five patients with severe mitral regurgitation caused by valve disease were excluded because the aortic valve area in these patients can be underestimated due to their low-flow state, even if the aortic valve is normal. Accordingly, the patient study group consisted of 120 patients (mean age 67 ± 13 years, 33-91 years, 97 males). CKD was defined as kidney damage or the presence of an estimated glomerular filtration rate (eGFR) < 60 ml/min/1.73 m^2 ^for 3 months or more, irrespective of cause [10,11]. The eGFR of each patient was calculated from their serum creatinine (SCr) value and their age using the following equation [12]: eGFR (ml/min/1.73 m^2^) = 194 × Age^-0.287 ^× SCr^-1.094 ^(if female × 0.739). Kidney damage was ascertained by the presence of albuminuria, defined as albumin-to-creatinine ratio > 30 mg/g in two of three spot urine specimens [10,11]. CKD was staged according to accepted NIH/NIDDK Kidney Disease Outcomes Quality Initiative standards [11]. The protocol was approved for use by the Human Studies Subcommittee of Mie University Graduate School of Medicine.

### Echocardiography

All patients underwent complete transthoracic echocardiography using a Vivid 7 system (GE-Vingmed, Horten, Norway) and an Aplio Artida ultrasound system (Toshiba Medical Systems Corp., Tokyo, Japan) within 2 months before or after coronary angiography. Arm-cuff blood pressure measurements were performed at the beginning of each echocardiographic study for all patients. The size of the aorta and left atrium, interventricular and left ventricular (LV) posterior wall thicknesses, LV end-diastolic and end-systolic dimensions, and fractional shortening were assessed from the parasternal long axis view [13]. The LV outflow tract (LVOT) diameter was measured in the parasternal long-axis view in mid-systole, and then converted to LVOT area (LVOT area = π × (0.5 × LVOT diameter)^2^) [14,15]. Doppler-derived stroke volume was calculated as the product of the LVOT area and the LVOT velocity time integral (VTI) obtained by pulsed Doppler [15]. The ratio of peak early diastolic transmitral flow velocity to peak early diastole mitral annular velocity (E') at the lateral site in the mitral annulus (E/E') was calculated as a Doppler parameter reflecting LV filling pressure [16,17]. Aortic valve area (AVA) was calculated by the continuity equation; AVA = (LVOT are × LVOT VTI)/trans-aortic VTI [18,19]. Severity of aortic stenosis (AS) was defined as follows: severe stenosis, AVA < 1.0 cm^2^; moderate stenosis, AVA 1.0 to 1.5 cm^2^; and mild stenosis, AVA 1.5 to 2.0 cm^2 ^[20]. AVA index, normalized by body surface area, was also calculated [20]. Peak velocity and mean pressure gradient across the aortic valve were also measured [20]. Aortic calcification was defined upon increased thickness and bright echoes of the valve leaflets. All Doppler values represent an average of 3 beats in the case of sinus rhythm and an average of 5 beats in the case of atrial fibrillation.

### Coronary Angiography

Coronary artery disease (CAD) was defined as a history of myocardial infarction, coronaryangioplasty, coronary artery bypass graft surgery, and a ≥ 50% luminal diameter stenosis in ≥ 1 epicardial coronary artery by quantitative coronary angiography. Selective catheter coronary angiography was performed via the transradial or transbrachial Judkins approach using standard techniques after nitroglycerin administration. Quantitative coronary angiography was performed using an automated edge-detection system (QAngioXA, MEDIS medical imaging systems, Leiden, the Netherlands) by a single observer unaware of the echocardiographic results. At least 2 orthogonal projections were evaluated, and measurement of percent diameter stenosis was performed in the projection showing the highest degree of narrowing.

### Statistical Analysis

Continuous variables were presented by mean and standard deviation and compared using a Student's t test or the Mann-Whitney's U test. Bonferroni correction was applied for multiple comparisons. Categorical variables were presented as percent frequencies and differences between proportions were compared using χ^2 ^test. P-values of less than 0.05 were considered to be statistically significant.

## Results

### Clinical characteristics

All patients underwent successful coronary angiography and transthoracic echocardiography. Of 120 patients, 2 patients (2%) had severe AS, 9 patients (8%) had moderate AS, and 30 patients (25%) had mild AS. Table 1 shows the clinical characteristics of the study subjects. Patients with AS were older, more often female, and had smaller body sizes than those without AS. The prevalence of coexisting hypertension, diabetes mellitus, and dyslipidemia was similar between the two patient groups, but patients with AS were less often categorized as current smokers than those without AS. Ninety-three of all 120 patients (78%) had CAD, and 56% of CAD patients had multi-vessel disease. The prevalence of CAD was similar between patients with and without AS. Of all 120 patients, 53% had CKD; 81% of these had stage 3 CKD (eGFR 59 to 30 ml/min/1.73 m^2^) and 19% had stage 4 CKD (eGFR 29 to 15 ml/min/1.73 m^2^). Patients with AS had a greater occurrence of CKD than those without AS. Patients with and without AS received similar medications. Although patients with AS had lower eGFR than those without AS, serum hemoglobin, calcium, and phosphate levels were similar between the two patients groups. Plasma lipid profile levels, hemoglobin A1C, and c-reactive protein were not significantly different between the two patient groups.

**Table 1:** Clinical Characteristics of Study Subjects

	Non-AS (n = 79)	AS (n = 41)
Demographics		
Mean age, years	65 ± 12	72 ± 12*
Female gender, %	11	34*
Height, cm	165 ± 8	157 ± 9*
Body weight, kg	65 ± 12	58 ± 10*
Systolic blood pressure, mmHg	129 ± 21	137 ± 28
Heart rate, beats/min	67 ± 12	69 ± 14
Medical history		
Hypertension, %	70	78
Diabetes mellitus, %	34	39
Dyslipidemia, %	67	71
Current smoking, %	46	20*
Coronary artery disease, %	73	85
Chronic kidney disease, %	41	76*
Medication use		
Beta blocker, %	27	29
Calcium channel blocker, %	39	44
ACEI/ARB, %	56	66
Diuretics, %	20	20
Statin, %	46	46
Measurements		
eGFR, ml/min/1.73 m^2^	64 ± 23	53 ± 18*
Hemoglobin, g/dl	13.2 ± 2.0	12.8 ± 1.9
Calcium, mg/dl	9.4 ± 0.6	9.5 ± 0.6
Phosphate, mg/dl	3.4 ± 0.6	3.4 ± 0.6
Total cholesterol, mg/dl	185 ± 44	182 ± 30
HDL-cholesterol, mg/dl	50 ± 18	50 ± 14
LDL-cholesterol, mg/dl	108 ± 36	105 ± 24
Triglyceride, mg/dl	135 ± 64	131 ± 55
Hemoglobin A1C, %	6.0 ± 1.0	6.1 ± 0.8
C-reactive protein, mg/dl	0.20 ± 0.28	0.32 ± 0.79

AS: aortic stenosis; ACEI: angiotensin converting enzyme inhibitor; ARB: angiotensin receptor blocker; eGFR: estimated glomerular filtration rate; HDL: high density lipoprotein; LDL: low density lipoprotein. *P < 0.05 versus non-AS.

### Echocardiographic Data

Table 2 shows the echcardiographic data from the study subjects. LV geometry and fractional shortening were similar between patients with and without AS. LVOT diameter and stroke volume were lower in patients with AS; however, stroke volume index was similar between patients with and without AS (33 ± 9 and 36 ± 8 ml/m^2^, p = ns). E/E' was similar between patients with and without AS. Aortic valve calcification was more frequent in patients with AS compared to those without AS. Peak velocity and the mean pressure gradient across the aortic valve during ejection were higher and AVA was smaller in patients with AS compared to those without AS.

**Table 2:** Echocardiographic Data of Study Subjects

	Non-AS (n = 79)	AS (n = 41)
Aortic diameter, mm	30 ± 3	29 ± 2
Left atrial diameter, mm	38 ± 6	39 ± 8
Interventricular septal thickness, mm	10 ± 2	10 ± 2
Posterior wall thickness, mm	10 ± 2	10 ± 2
LV end-diastolic diameter, mm	50 ± 8	48 ± 7
LV end-systolic diameter, mm	36 ± 10	34 ± 9
Fractional shortening, %	30 ± 10	30 ± 9
LV outflow diameter, mm	21 ± 1	20 ± 2*
Stroke volume, ml	60 ± 12	52 ± 15*
E/E'	9 ± 4	11 ± 6
Aortic valve calcification, %	30	68*
Peak velocity across the aortic valve, m/s	1.27 ± 0.21	1.70 ± 0.41*
Mean pressure gradient across the aortic valve, mmHg	3.53 ± 1.17	6.20 ± 3.28*
Aortic valve area, cm^2^	2.58 ± 0.39	1.62 ± 0.33*

AS: aortic stenosis; LV: left ventricular; E/E': ratio of peak early diastolic transmitral flow velocity to peak early diastole mitral annular velocity. *P < 0.05 versus non-AS.

### Determinants of AVA Index

**Figure 1:**
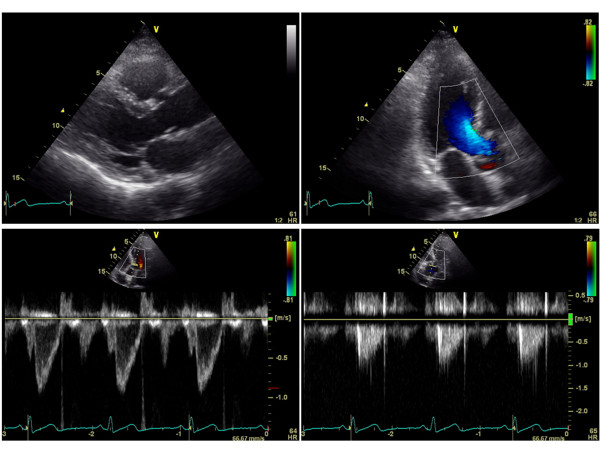
**Two-dimentioal and Doppler echocardiographic images from a patient without CKD or CAD**. CKD: chronic kidney disease; CAD: coronary artery disease.

**Figure 2:**
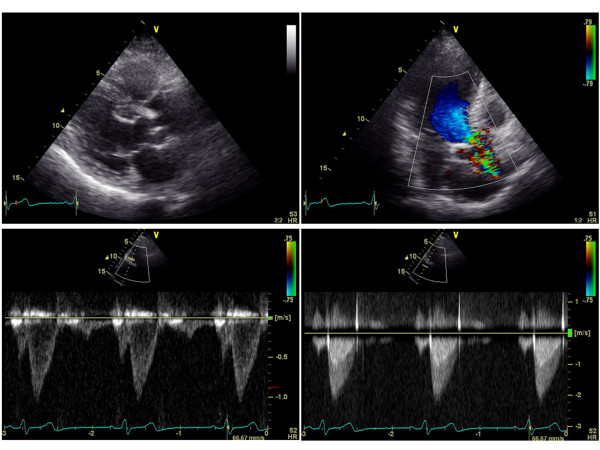
**Two-dimentioal and Doppler echocardiographic images from a patient with both CKD and CAD**. CKD: chronic kidney disease; CAD: coronary artery disease.

**Figure 3:**
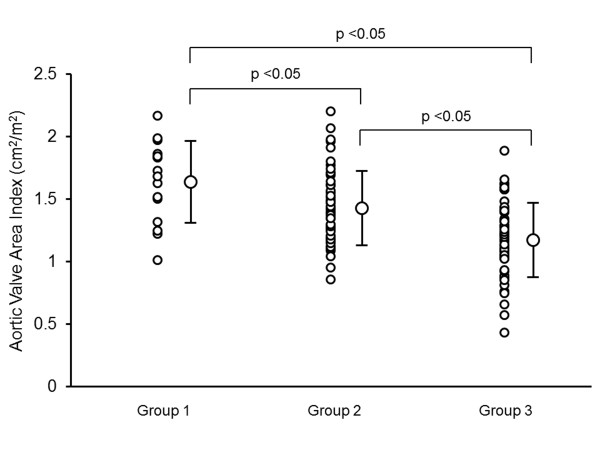
**Plots showing aortic valve area index in group 1 (absence of CKD and CAD, n = 16), group 2 (presence of either CKD or CAD, n = 51), and group 3 (presence of both CKD and CAD, n = 53)**. CKD: chronic kidney disease; CAD: coronary artery disease.

**Figure 4:**
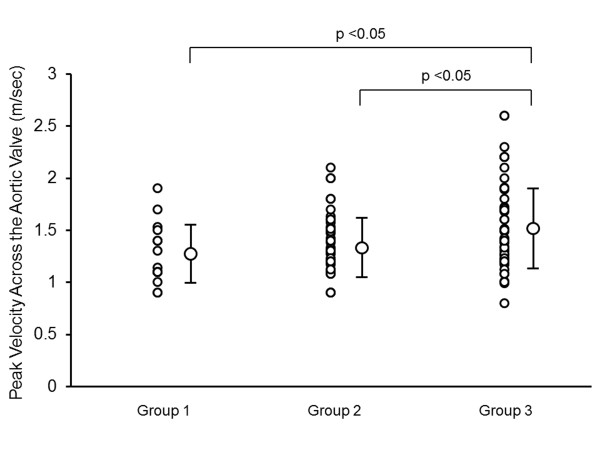
**Plots showing peak velocity across the aortic valve in group 1 (absence of CKD and CAD, n = 16), group 2 (presence of either CKD or CAD, n = 51), and group 3 (presence of both CKD and CAD, n = 53)**. CKD: chronic kidney disease; CAD: coronary artery disease.

Multivariate linear regression analysis was used to identify clinical (age, gender, blood pressures, presences of hypertension, diabetes mellitus, dyslipidemia, current smoking, CAD, CKD, medication uses, and laboratory measurements) and echocardiographic variables that might predict AVA index with a stepwise method. The results indicated that only the presence of CKD and CAD were independent determinants of AVA index with standardized coefficients of -0.37 and -0.28, respectively. Figures 1 and 2 show typical examples of 2-dimentioal and Doppler echocardiographic appearances of the aortic valve in a patient without CKD or CAD, and a patient with both CKD and CAD, respectively. The patient without CKD or CAD (62 year-old male, eGFR: 78 ml/min/1.73 m^2^) had non-calcified aortic valve with calculated AVA of 3.2 cm^2 ^(Figure 1). In contrast, the patient with both CKD and CAD (77 year-old male, eGFR: 48 ml/min/1.73 m^2^, 1 vessel disease) had a restricted aortic valve with calculated AVA of 1.7 cm^2 ^and a turbulent flow across the valve with peak velocity of 2.2 m/sec (Figure 2). When dividing patients into 3 groups according to the presence or absence of CKD and CAD (group 1: absence of CKD and CAD; group 2: presence of either CKD or CAD; group 3: presence of both CKD and CAD), group 2 had significantly lower AVA index than group 1, and group 3 had significantly lower AVA index than both groups 1 and 2 (group 1: 1.65 ± 0.32 cm^2^/m^2^; group 2: 1.43 ± 0.29* cm^2^/m^2^; and group 3: 1.19 ± 0.30*# cm^2^/m^2^; *p < 0.05 vs. group 1, and #p < 0.05 vs. group 2, respectively; Figure 3). Peak velocity across the aortic valve was significantly higher in the group 3 than both groups 1 and 2 (group 1: 1.28 ± 0.29 m/sec; group 2: 1.35 ± 0.27 m/sec; and group 3: 1.53 ± 0.41*#m/sec; *p < 0.05 vs. group 1, and #p < 0.05 vs. group 2, respectively; Figure 4).

## Discussion

We quantified the AVA index by transthoracic echocardiography in consecutive patients receiving coronary angiography and clarified that CKD, even pre-stage 5 CKD, was the most powerful determinant of AVA index among a high risk population for coronary atherosclerosis.

Calcific aortic valve disease is frequent in the general population and is associated with high cardiovascular risk [1,2]. This valvular disease is a slowly progressive disorder with a disease continuum that ranges from mild valve thickening without obstruction of blood flow, termed aortic sclerosis, to severe calcification with impaired leaflet motion, or AS [21]. The pathologic mechanisms involved in the disease include active processes that are similar to those occurring in coronary atherosclerosis, such as the impairment of endothelium, inflammation, and lipid infiltration [21-23]. Thus, calcific aortic valve disease has been widely shown to correlate with the presence and severity of CAD [3,24,25].

Despite the similarities in the histopathologic features and clinical factors associated with calcific aortic valve disease and coronary atherosclerosis, however, discrepancies also exist. For example, although calcific changes can be seen in the atherosclerotic plaques of coronary arteries, calcification occurs earlier and is a more prominent feature of calcific aortic valve disease, particularly in the end stages of the disease process, and a large contributor of disease progression is prominent calcification with a gradual increase in leaflet thickness and outflow obstruction [26]. The importance of tissue calcification in the disease process is highlighted by the observation that subsets of patients with altered mineral metabolism such as CKD have a higher prevalence of calcific aortic valve disease and more rapid disease progression [6-9]. However, most previous studies have demonstrated an association between end-stage renal disease and calcific cardiovascular disease, especially in patients on dialysis [6-9], and therefore, less is known about the impact of early stage CKD on the prevalence and severity of calcific aortic valve disease. The present study indicates that patients with early stage CKD have an increased prevalence of AS in the populations at high risk for coronary atherosclerosis. Notably, most of patients with CKD in the present study were in stage 3, and mean eGFR in patients with and without AS was 64 ± 23 and 53 ± 18 ml/min/1.73 m^2^, respectively, suggesting that small differences in renal function in early stages CKD can contribute to the progression of calcific aortic valve disease in patients at high risk for coronary atherosclerosis. Although it would be difficult to address the underlying mechanisms that are responsible for the progression of calcific aortic valve disease in these earlier stages of CKD, the present study suggests that latent alterations in multiple factors such as inflammation, anemia, oxidative stress, abnormal calcium/phosphate metabolism, and hemodynamic overload may interdependently contribute to the disease process, even when serum calcium/phosphate levels are within normal ranges, and c-reactive protein levels, hemoglobin levels, and echocardiographic parameters of LV filling pressure are statistically similar between patients with and without AS. Although the present study did not focus on activation of proinflammatory mechanisms as a causal mechanism for the progression of calcific aortic valve disease, Aikawa et al demonstrated that proinflammatory cathepsin S, a highly potent elastase, contributes to arterial and valvular calcification in mice with atherosclerosis and CKD assessed by in vivo and ex vivo optical molecular imaging [27]. They used molecular imaging of early calcification and elastolytic activity to address new mechanisms underlying accelerated calcification in patients with CKD and suggested that calcification is a multifactorial process induced by proinflammatory stimuli that promote accumulation of elastolytic macrophages. Although the present study did not focus on abnormalities in mineral metabolism as a causal mechanism for the progression of calcific aortic valve disease, previous clinical studies have demonstrated that a decrease in 1,25-dihydroxyvitamin D and an increase in parathyroid hormone (PTH) are the earliest mineral metabolic events that take place in CKD, while serum calcium and phosphate levels are altered later in the course of CKD [28]. Adeney et al. demonstrated that higher serum phosphate concentrations within the normal laboratory range were associated with a statistically greater prevalence of coronary artery, descending thoracic aorta, and mitral valve calcification in a community-based cohort of individuals with moderate CKD and no clinically apparent cardiovascular disease [29]. A decline in renal function leads to phosphate retention, elevated PTH levels, and low 1,25-dihydroxy vitamin D levels; however, serum phosphate levels are often maintained within the normal laboratory range until relatively late in the course of CKD [30-32]. Therefore, evaluation of the levels of serum PTH and 1,25-dihydroxy vitamin D, early markers for impaired mineral metabolism, is important to clarify the underlying mechanisms that are responsible for the progression of calcific aortic valve disease in early stage CKD.

Several studies have documented an overlap in the clinical factors traditionally associated with calcific valve disease and coronary atherosclerosis [33-38]. In the prospective population-based Cardiovascular Health Study, which included 5621 adults over the age of 65 years, clinical factors associated with calcific aortic valve disease included age, gender, smoking, hypertension, and hyperlipidemia [33]. However, CKD was not included as a potential contributor to aortic valve calcification and stenosis in these studies. In contrast, our study demonstrated that conventional risk factors for cardiovascular atherosclerosis were not independently related to the progression of aortic valve disease, possibly because they were well-controlled. Despite receiving adequate risk factor management, more than half of the patients developed CKD in the present study, and CKD, even pre-stage 5 CKD, has a more powerful impact on the presence and severity of AS than other conventional risk factors for atherosclerosis in patients at high risk for CAD. These results indicate that patients with CKD should be included as part of the highest risk group for the progression of calcific aortic valve disease. Aortic valve calcification impairs the movement of aortic valve leaflets, which affects cardiac function, and can only be alleviated through costly and invasive procedures. Therefore, early diagnosis of and interference with aortic valve calcification could provide enormous clinical benefits.

Although 34% of patients were diagnosed with AS based on AVA, the average value of their peak velocities and mean pressure gradients across the aortic valve were only 1.70 ± 0.41 m/sec and 6.20 ± 3.28 mmHg, respectively. Patients with AS in our study population were older, had smaller body size, were more often women, and had smaller LVOT diameter compared with those without AS, which may partially be associated with low-flow and low-gradient AS [39]. Nevertheless, the present study demonstrated that presence of both with CKD and CAD had a great impact on reduced AVA index and elevated peak velocity across the aortic valve.

A potential limitation of the present study was the small sample population, although we succeeded in testing our primary hypothesis, which showed the impact of CKD on the presence and the severity of AS in patients at high risk for CAD. Three dimensional measurements of LVOT and aortic valve area were not involved in this study. However, Doppler-derived estimation of AVA using two-dimensional transthoracic echocardiography is considered to correlate well with simultaneous catheter-derived measurements, and is widely used clinically [14-16]. Invasive pressure measurements were not involved in the present study; therefore, evaluation of AVA using Gorlin's formula was not possible. Also, direct measures of LV end-diastolic pressure or pulmonary wedge pressure were not possible. Accordingly, E/E' was recruited as a marker of LV filling pressure in the present study [17,18]. Finally, evaluation of the rate of progression of aortic stenosis or the clinical outcome was not included in our study. The long-term effect of CKD on the progression of calcific aortic valve disease warrants further investigation.

## List of abbreviations

CKD: chronic kidney disease; AS: aortic stenosis; CAD: coronary artery disease; AVA: aortic valve area; eGFR: estimated glomerular filtration rate; SCr: serum creatinine; LV: left ventricular; LVOT: outflow tract; VTI: velocity time integral; E': peak early diastole mitral annular velocity; E/E': The ratio of peak early diastolic transmitral flow velocity to peak early diastole mitral annular velocity; PTH: parathyroid hormone.

## Competing interests

The authors declare that they have no competing interests.

## Authors' contributions

CM, YS, YB, HF, and SF acquired the ultrasound images and performed the analysis. CM and KD contributed substantially to data interpretation and wrote the manuscript. KD contributed substantially to the conception and design of the study, and critical revision of the manuscript for important intellectual content. TS and MS assisted in data interpretation. KO and KS contributed to critical revision of the manuscript for important intellectual content and study supervision. MI and TN reviewed the manuscript. Finally, all authors read and approved the manuscript.
